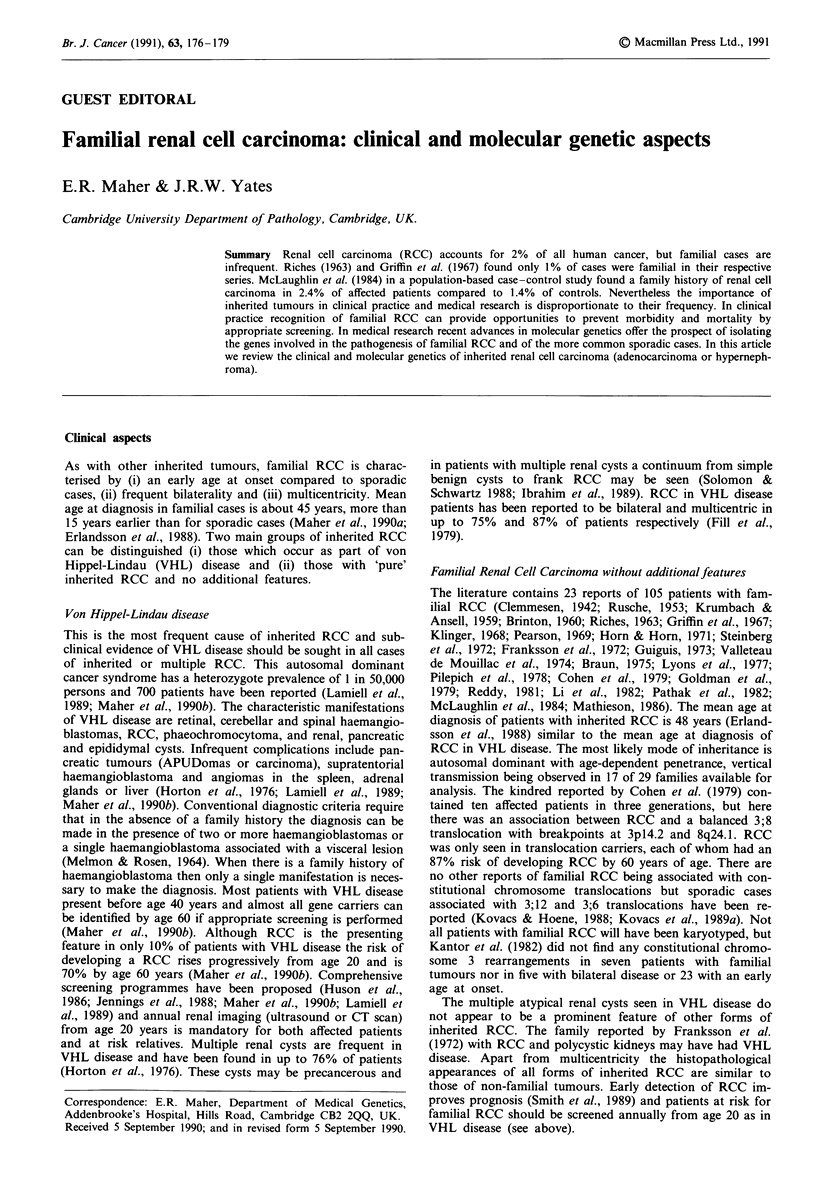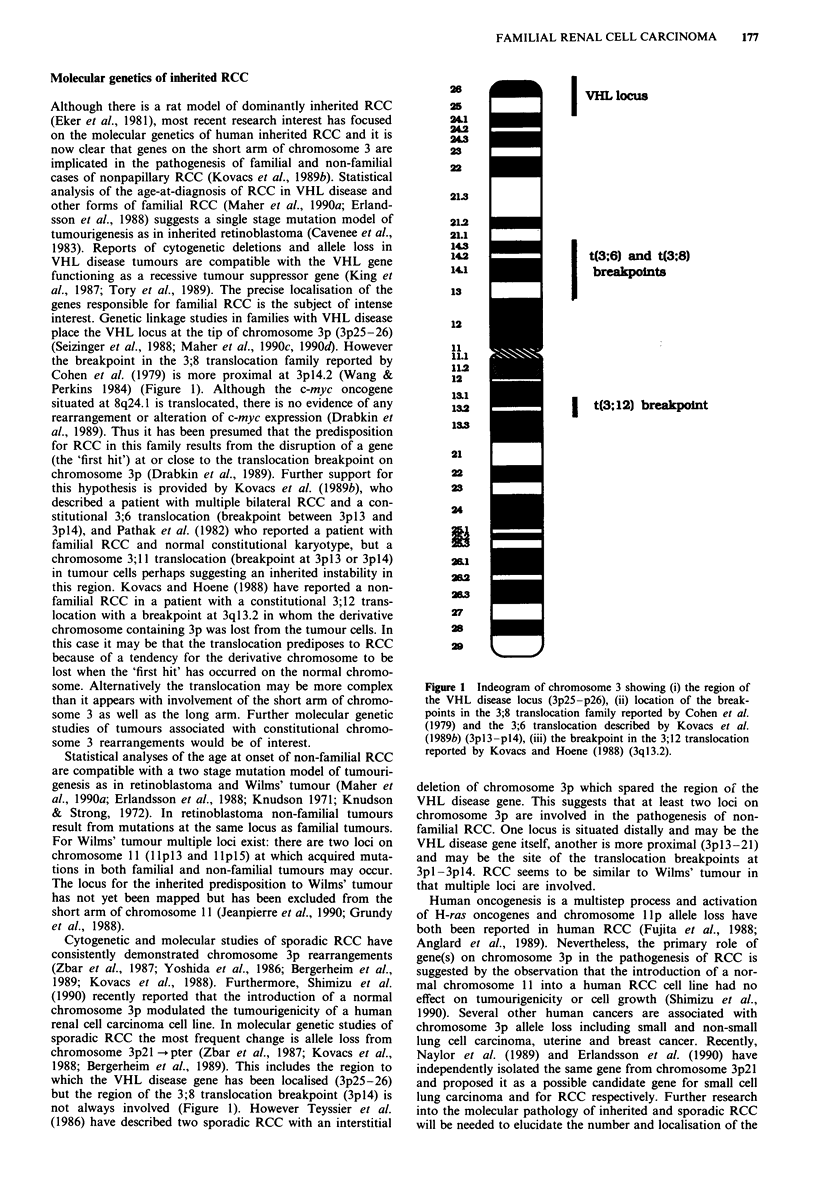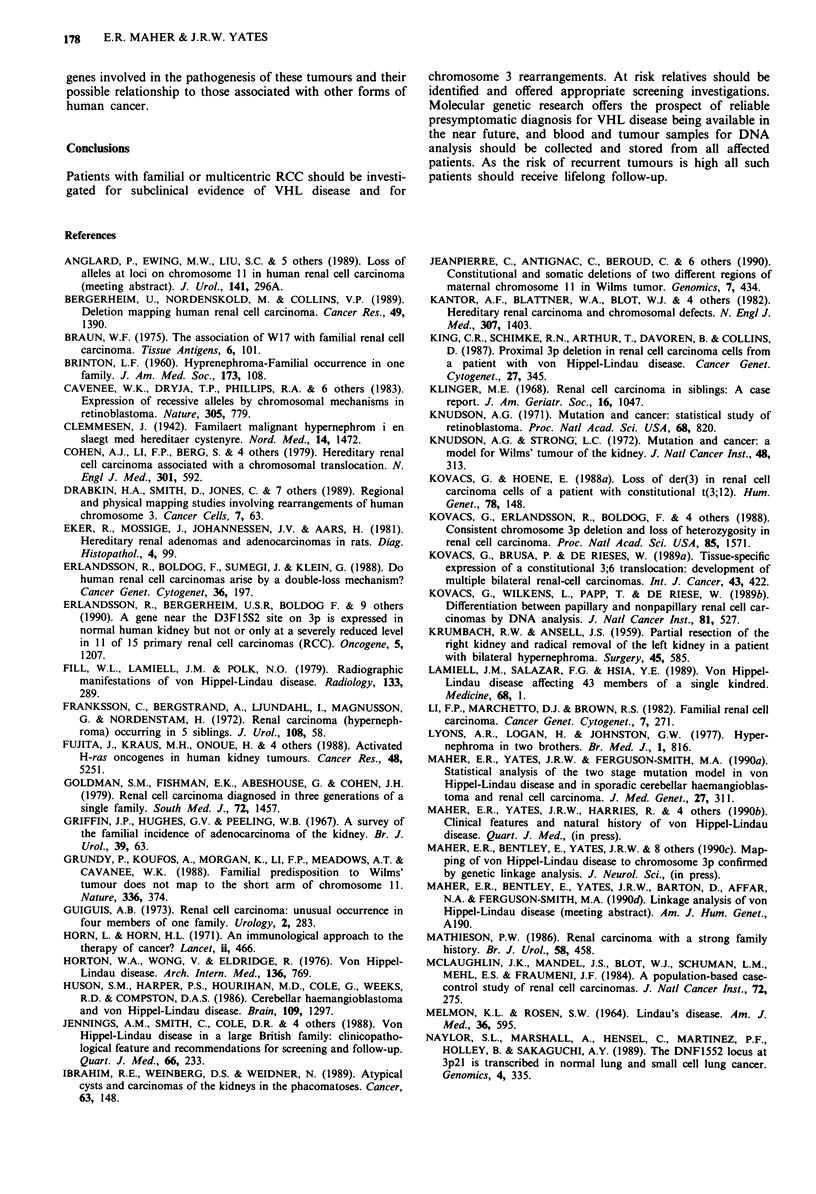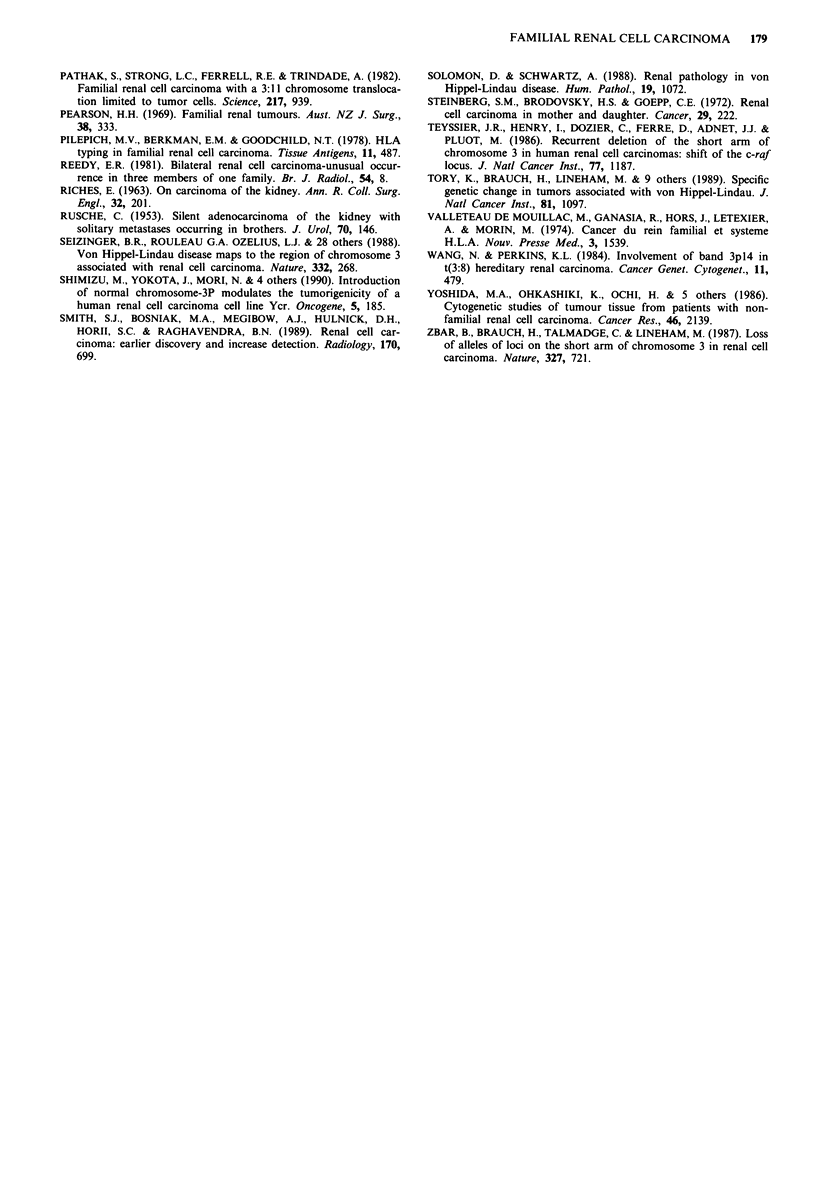# Familial renal cell carcinoma: clinical and molecular genetic aspects

**DOI:** 10.1038/bjc.1991.43

**Published:** 1991-02

**Authors:** E.R. Maher, J.R.W. Yates

## Abstract

Renal cell carcinoma (RCC) accounts for 2% of all human cancer, but familial cases are infrequent. Riches (1963) and Griffin *et al.* (1984) in a population-based case-control study found a family history of renal cell carcinoma in 2.4% of affected patients compared to 1.4% of controls. Nevertheless the importance of inherited tumours in clinical practice and medical research is disproportionate to their frequency. In clinical practice recognition of familial RCC can provide opportunities to prevent morbidity and mortality by appropriate screening. In medical research recent advances in molecular genetics offer the prospect of isolating the genes involved in the pathogenesis of familial RCC and of the more common sporadic cases. In this article we review the clinical and molecular genetics of inherited renal cell carcinoma (adenocarcinoma or hypernephroma).


Br. J. Cancer (1991), 63, 176–179 © Macmillan Press Ltd., 1991

GUEST EDITORAL

### Familial renal cell carcinoma: clinical and molecular genetic aspects

E.R. Maher & J.R.W. Yates

*Cambridge University Department of Pathology, Cambridge, UK.*

**Summary** Renal cell carcinoma (RCC) accounts for 2% of all human cancer, but familial cases are infrequent. Riches (1963) and Griffin *et al.* (1967) found only 1% of cases were familial in their respective series. McLaughlin *et al.* (1984) in a population-based case–control study found a family history of renal cell carcinoma in 2.4% of affected patients compared to 1.4% of controls. Nevertheless the importance of inherited tumours in clinical practice and medical research is disproportionate to their frequency. In clinical practice recognition of familial RCC can provide opportunities to prevent morbidity and mortality by appropriate screening. In medical research recent advances in molecular genetics offer the prospect of isolating the genes involved in the pathogenesis of familial RCC and of the more common sporadic cases. In this article we review the clinical and molecular genetics of inherited renal cell carcinoma (adenocarcinoma or hypernephroma).

### Clinical aspects

As with other inherited tumours, familial RCC is characterised by (i) an early age at onset compared to sporadic cases, (ii) frequent bilaterality and (iii) multicentricity. Mean age at diagnosis in familial cases is about 45 years, more than 15 years earlier than for sporadic cases (Maher *et al.*, 1990*a*; Erlandsson *et al.*, 1988). Two main groups of inherited RCC can be distinguished (i) those which occur as part of von Hippel-Lindau (VHL) disease and (ii) those with 'pure' inherited RCC and no additional features.

### *Von Hippel-Lindau disease*

This is the most frequent cause of inherited RCC and subclinical evidence of VHL disease should be sought in all cases of inherited or multiple RCC. This autosomal dominant cancer syndrome has a heterozygote prevalence of 1 in 50,000 persons and 700 patients have been reported (Lamiell *et al.*, 1989; Maher *et al.*, 1990*b*). The characteristic manifestations of VHL disease are retinal, cerebellar and spinal haemangioblastomas, RCC, phaeochromocytoma, and renal, pancreatic and epididymal cysts. Infrequent complications include pancreatic tumours (APUDomas or carcinoma), supratentorial haemangioblastoma and angiomas in the spleen, adrenal glands or liver (Horton *et al.*, 1976; Lamiell *et al.*, 1989; Maher *et al.*, 1990*b*). Conventional diagnostic criteria require that in the absence of a family history the diagnosis can be made in the presence of two or more haemangioblastomas or a single haemangioblastoma associated with a visceral lesion (Melmon & Rosen, 1964). When there is a family history of haemangioblastoma then only a single manifestation is necessary to make the diagnosis. Most patients with VHL disease present before age 40 years and almost all gene carriers can be identified by age 60 if appropriate screening is performed (Maher *et al.*, 1990*b*). Although RCC is the presenting feature in only 10% of patients with VHL disease the risk of developing a RCC rises progressively from age 20 and is 70% by age 60 years (Maher *et al.*, 1990*b*). Comprehensive screening programmes have been proposed (Huson *et al.*, 1986; Jennings *et al.*, 1988; Maher *et al.*, 1990*b*; Lamiell *et al.*, 1989) and annual renal imaging (ultrasound or CT scan) from age 20 years is mandatory for both affected patients and at risk relatives. Multiple renal cysts are frequent in VHL disease and have been found in up to 76% of patients (Horton *et al.*, 1976). These cysts may be precancerous and in patients with multiple renal cysts a continuum from simple benign cysts to frank RCC may be seen (Solomon & Schwartz 1988; Ibrahim *et al.*, 1989). RCC in VHL disease patients has been reported to be bilateral and multicentric in up to 75% and 87% of patients respectively (Fill *et al.*, 1979).

Correspondence: E.R. Maher, Department of Medical Genetics, Addenbrooke's Hospital, Hills Road, Cambridge CB2 2QQ, UK.
Received 5 September 1990; and in revised form 5 September 1990.

### *Familial Renal Cell Carcinoma without additional features*

The literature contains 23 reports of 105 patients with familial RCC (Clemmesen, 1942; Rusche, 1953; Krumbach & Ansell, 1959; Brinton, 1960; Riches, 1963; Griffin *et al.*, 1967; Klinger, 1968; Pearson, 1969; Horn & Horn, 1971; Steinberg *et al.*, 1972; Franksson *et al.*, 1972; Guiguis, 1973; Valleteau de Mouillac *et al.*, 1974; Braun, 1975; Lyons *et al.*, 1977; Pilepich *et al.*, 1978; Cohen *et al.*, 1979; Goldman *et al.*, 1979; Reddy, 1981; Li *et al.*, 1982; Pathak *et al.*, 1982; McLaughlin *et al.*, 1984; Mathieson, 1986). The mean age at diagnosis of patients with inherited RCC is 48 years (Erlandsson *et al.*, 1988) similar to the mean age at diagnosis of RCC in VHL disease. The most likely mode of inheritance is autosomal dominant with age-dependent penetrance, vertical transmission being observed in 17 of 29 families available for analysis. The kindred reported by Cohen *et al.* (1979) contained ten affected patients in three generations, but here there was an association between RCC and a balanced 3;8 translocation with breakpoints at 3p14.2 and 8q24.1. RCC was only seen in translocation carriers, each of whom had an 87% risk of developing RCC by 60 years of age. There are no other reports of familial RCC being associated with constitutional chromosome translocations but sporadic cases associated with 3;12 and 3;6 translocations have been reported (Kovacs & Hoene, 1988; Kovacs *et al.*, 1989*a*). Not all patients with familial RCC will have been karyotyped, but Kantor *et al.* (1982) did not find any constitutional chromosome 3 rearrangements in seven patients with familial tumours nor in five with bilateral disease or 23 with an early age at onset.

The multiple atypical renal cysts seen in VHL disease do not appear to be a prominent feature of other forms of inherited RCC. The family reported by Franksson *et al.* (1972) with RCC and polycystic kidneys may have had VHL disease. Apart from multicentricity the histopathological appearances of all forms of inherited RCC are similar to those of non-familial tumours. Early detection of RCC improves prognosis (Smith *et al.*, 1989) and patients at risk for familial RCC should be screened annually from age 20 as in VHL disease (see above).

### Molecular genetics of inherited RCC

Although there is a rat model of dominantly inherited RCC (Eker *et al.*, 1981), most recent research interest has focused on the molecular genetics of human inherited RCC and it is now clear that genes on the short arm of chromosome 3 are implicated in the pathogenesis of familial and non-familial cases of nonpapillary RCC (Kovacs *et al.*, 1989*b*). Statistical analysis of the age-at-diagnosis of RCC in VHL disease and other forms of familial RCC (Maher *et al.*, 1990*a*; Erlandsson *et al.*, 1988) suggests a single stage mutation model of tumourigenesis as in inherited retinoblastoma (Cavenee *et al.*, 1983). Reports of cytogenetic deletions and allele loss in VHL disease tumours are compatible with the VHL gene functioning as a recessive tumour suppressor gene (King *et al.*, 1987; Tory *et al.*, 1989). The precise localisation of the genes responsible for familial RCC is the subject of intense interest. Genetic linkage studies in families with VHL disease place the VHL locus at the tip of chromosome 3p (3p25–26) (Seizinger *et al.*, 1988; Maher *et al.*, 1990*c*, 1990*d*). However the breakpoint in the 3;8 translocation family reported by Cohen *et al.* (1979) is more proximal at 3p14.2 (Wang & Perkins 1984) (Figure 1). Although the c-*myc* oncogene situated at 8q24.1 is translocated, there is no evidence of any rearrangement or alteration of c-*myc* expression (Drabkin *et al.*, 1989). Thus it has been presumed that the predisposition for RCC in this family results from the disruption of a gene (the 'first hit') at or close to the translocation breakpoint on chromosome 3p (Drabkin *et al.*, 1989). Further support for this hypothesis is provided by Kovacs *et al.* (1989*b*), who described a patient with multiple bilateral RCC and a constitutional 3;6 translocation (breakpoint between 3p13 and 3p14), and Pathak *et al.* (1982) who reported a patient with familial RCC and normal constitutional karyotype, but a chromosome 3;11 translocation (breakpoint at 3p13 or 3p14) in tumour cells perhaps suggesting an inherited instability in this region. Kovacs and Hoene (1988) have reported a non-familial RCC in a patient with a constitutional 3;12 translocation with a breakpoint at 3q13.2 in whom the derivative chromosome containing 3p was lost from the tumour cells. In this case it may be that the translocation prediposes to RCC because of a tendency for the derivative chromosome to be lost when the 'first hit' has occurred on the normal chromosome. Alternatively the translocation may be more complex than it appears with involvement of the short arm of chromosome 3 as well as the long arm. Further molecular genetic studies of tumours associated with constitutional chromosome 3 rearrangements would be of interest.

Statistical analyses of the age at onset of non-familial RCC are compatible with a two stage mutation model of tumourigenesis as in retinoblastoma and Wilms' tumour (Maher *et al.*, 1990*a*; Erlandsson *et al.*, 1988; Knudson 1971; Knudson & Strong, 1972). In retinoblastoma non-familial tumours result from mutations at the same locus as familial tumours. For Wilms' tumour multiple loci exist: there are two loci on chromosome 11 (11p13 and 11p15) at which acquired mutations in both familial and non-familial tumours may occur. The locus for the inherited predisposition to Wilms' tumour has not yet been mapped but has been excluded from the short arm of chromosome 11 (Jeanpierre *et al.*, 1990; Grundy *et al.*, 1988).

Cytogenetic and molecular studies of sporadic RCC have consistently demonstrated chromosome 3p rearrangements (Zbar *et al.*, 1987; Yoshida *et al.*, 1986; Bergerheim *et al.*, 1989; Kovacs *et al.*, 1988). Furthermore, Shimizu *et al.* (1990) recently reported that the introduction of a normal chromosome 3p modulated the tumourigenicity of a human renal cell carcinoma cell line. In molecular genetic studies of sporadic RCC the most frequent change is allele loss from chromosome 3p21 $\rightarrow$ pter (Zbar *et al.*, 1987; Kovacs *et al.*, 1988; Bergerheim *et al.*, 1989). This includes the region to which the VHL disease gene has been localised (3p25–26) but the region of the 3;8 translocation breakpoint (3p14) is not always involved (Figure 1). However Teyssier *et al.* (1986) have described two sporadic RCC with an interstitial

**Figure 1** Indeogram of chromosome 3 showing (i) the region of the VHL disease locus (3p25–p26), (ii) location of the breakpoints in the 3;8 translocation family reported by Cohen *et al.* (1979) and the 3;6 translocation described by Kovacs *et al.* (1989*b*) (3p13–p14), (iii) the breakpoint in the 3;12 translocation reported by Kovacs and Hoene (1988) (3q13.2).

deletion of chromosome 3p which spared the region of the VHL disease gene. This suggests that at least two loci on chromosome 3p are involved in the pathogenesis of non-familial RCC. One locus is situated distally and may be the VHL disease gene itself, another is more proximal (3p13–21) and may be the site of the translocation breakpoints at 3p1–3p14. RCC seems to be similar to Wilms' tumour in that multiple loci are involved.

Human oncogenesis is a multistep process and activation of H-*ras* oncogenes and chromosome 11p allele loss have both been reported in human RCC (Fujita *et al.*, 1988; Anglard *et al.*, 1989). Nevertheless, the primary role of gene(s) on chromosome 3p in the pathogenesis of RCC is suggested by the observation that the introduction of a normal chromosome 11 into a human RCC cell line had no effect on tumourigenicity or cell growth (Shimizu *et al.*, 1990). Several other human cancers are associated with chromosome 3p allele loss including small and non-small lung cell carcinoma, uterine and breast cancer. Recently, Naylor *et al.* (1989) and Erlandsson *et al.* (1990) have independently isolated the same gene from chromosome 3p21 and proposed it as a possible candidate gene for small cell lung carcinoma and for RCC respectively. Further research into the molecular pathology of inherited and sporadic RCC will be needed to elucidate the number and localisation of the genes involved in the pathogenesis of these tumours and their possible relationship to those associated with other forms of human cancer.

### Conclusions

Patients with familial or multicentric RCC should be investigated for subclinical evidence of VHL disease and for chromosome 3 rearrangements. At risk relatives should be identified and offered appropriate screening investigations. Molecular genetic research offers the prospect of reliable presymptomatic diagnosis for VHL disease being available in the near future, and blood and tumour samples for DNA analysis should be collected and stored from all affected patients. As the risk of recurrent tumours is high all such patients should receive lifelong follow-up.

### References

ANGLARD, P., EWING, M.W., LIU, S.C. & 5 others (1989). Loss of alleles at loci on chromosome 11 in human renal cell carcinoma (meeting abstract). *J. Urol.*, **141**, 296A.

BERGERHEIM, U., NORDENSKOLD, M. & COLLINS, V.P. (1989). Deletion mapping human renal cell carcinoma. *Cancer Res.*, **49**, 1390.

BRAUN, W.F. (1975). The association of W17 with familial renal cell carcinoma. *Tissue Antigens*, **6**, 101.

BRINTON, L.F. (1960). Hyprenephroma-Familial occurrence in one family. *J. Am. Med. Soc.*, **173**, 108.

CAVENEE, W.K., DRYJA, T.P., PHILLIPS, R.A. & 6 others (1983). Expression of recessive alleles by chromosomal mechanisms in retinoblastoma. *Nature*, **305**, 779.

CLEMMESEN, J. (1942). Familaert malignant hypernephrom i en slaegt med hereditaer cystenyre. *Nord. Med.*, **14**, 1472.

COHEN, A.J., LI, F.P., BERG, S. & 4 others (1979). Hereditary renal cell carcinoma associated with a chromosomal translocation. *N. Engl J. Med.*, **301**, 592.

DRABKIN, H.A., SMITH, D., JONES, C. & 7 others (1989). Regional and physical mapping studies involving rearrangements of human chromosome 3. *Cancer Cells*, **7**, 63.

EKER, R., MOSSIGE, J., JOHANNESSEN, J.V. & AARS, H. (1981). Hereditary renal adenomas and adenocarcinomas in rats. *Diag. Histopathol.*, **4**, 99.

ERLANDSSON, R., BOLDOG, F., SUMEGI, J. & KLEIN, G. (1988). Do human renal cell carcinomas arise by a double-loss mechanism? *Cancer Genet. Cytogenet*, **36**, 197.

ERLANDSSON, R., BERGERHEIM, U.S.R, BOLDOG F. & 9 others (1990). A gene near the D3F15S2 site on 3p is expressed in normal human kidney but not or only at a severely reduced level in 11 of 15 primary renal cell carcinomas (RCC). *Oncogene*, **5**, 1207.

FILL, W.L., LAMIELL, J.M. & POLK, N.O. (1979). Radiographic manifestations of von Hippel-Lindau disease. *Radiology*, **133**, 289.

FRANKSSON, C., BERGSTRAND, A., LJUNDAHL, I., MAGNUSSON, G. & NORDENSTAM, H. (1972). Renal carcinoma (hypernephroma) occurring in 5 siblings. *J. Urol.*, **108**, 58.

FUJITA, J., KRAUS, M.H., ONOUE, H. & 4 others (1988). Activated H-*ras* oncogenes in human kidney tumours. *Cancer Res.*, **48**, 5251.

GOLDMAN, S.M., FISHMAN, E.K., ABESHOUSE, G. & COHEN, J.H. (1979). Renal cell carcinoma diagnosed in three generations of a single family. *South Med. J.*, **72**, 1457.

GRIFFIN, J.P., HUGHES, G.V. & PEELING, W.B. (1967). A survey of the familial incidence of adenocarcinoma of the kidney. *Br. J. Urol.*, **39**, 63.

GRUNDY, P., KOUFOS, A., MORGAN, K., LI, F.P., MEADOWS, A.T. & CAVANEE, W.K. (1988). Familial predisposition to Wilms' tumour does not map to the short arm of chromosome 11. *Nature*, **336**, 374.

GUIGUIS, A.B. (1973). Renal cell carcinoma: unusual occurrence in four members of one family. *Urology*, **2**, 283.

HORN, L. & HORN, H.L. (1971). An immunological approach to the therapy of cancer? *Lancet*, **ii**, 466.

HORTON, W.A., WONG, V. & ELDRIDGE, R. (1976). Von Hippel-Lindau disease. *Arch. Intern. Med.*, **136**, 769.

HUSON, S.M., HARPER, P.S., HOURIHAN, M.D., COLE, G., WEEKS, R.D. & COMPSTON, D.A.S. (1986). Cerebellar haemangioblastoma and von Hippel-Lindau disease. *Brain*, **109**, 1297.

JENNINGS, A.M., SMITH, C., COLE, D.R. & 4 others (1988). Von Hippel-Lindau disease in a large British family: clinicopathological feature and recommendations for screening and follow-up. *Quart. J. Med.*, **66**, 233.

IBRAHIM, R.E., WEINBERG, D.S. & WEIDNER, N. (1989). Atypical cysts and carcinomas of the kidneys in the phacomatoses. *Cancer*, **63**, 148.

JEANPIERRE, C., ANTIGNAC, C., BEROUD, C. & 6 others (1990). Constitutional and somatic deletions of two different regions of maternal chromosome 11 in Wilms tumor. *Genomics*, **7**, 434.

KANTOR, A.F., BLATTNER, W.A., BLOT, W.J. & 4 others (1982). Hereditary renal carcinoma and chromosomal defects. *N. Engl J. Med.*, **307**, 1403.

KING, C.R., SCHIMKE, R.N., ARTHUR, T., DAVOREN, B. & COLLINS, D. (1987). Proximal 3p deletion in renal cell carcinoma cells from a patient with von Hippel-Lindau disease. *Cancer Genet. Cytogenet.*, **27**, 345.

KLINGER, M.E. (1968). Renal cell carcinoma in siblings: A case report. *J. Am. Geriatr. Soc.*, **16**, 1047.

KNUDSON, A.G. (1971). Mutation and cancer: statistical study of retinoblastoma. *Proc. Natl Acad. Sci. USA*, **68**, 820.

KNUDSON, A.G. & STRONG, L.C. (1972). Mutation and cancer: a model for Wilms' tumour of the kidney. *J. Natl Cancer Inst.*, **48**, 313.

KOVACS, G. & HOENE, E. (1988*a*). Loss of der(3) in renal cell carcinoma cells of a patient with constitutional t(3;12). *Hum. Genet.*, **78**, 148.

KOVACS, G., ERLANDSSON, R., BOLDOG, F. & 4 others (1988). Consistent chromosome 3p deletion and loss of heterozygosity in renal cell carcinoma. *Proc. Natl Acad. Sci. USA*, **85**, 1571.

KOVACS, G., BRUSA, P. & DE RIESES, W. (1989*a*). Tissue-specific expression of a constitutional 3;6 translocation: development of multiple bilateral renal-cell carcinomas. *Int. J. Cancer*, **43**, 422.

KOVACS, G., WILKENS, L., PAPP, T. & DE RIESE, W. (1989*b*). Differentiation between papillary and nonpapillary renal cell carcinomas by DNA analysis. *J. Natl Cancer Inst.*, **81**, 527.

KRUMBACH, R.W. & ANSELL, J.S. (1959). Partial resection of the right kidney and radical removal of the left kidney in a patient with bilateral hypernephroma. *Surgery*, **45**, 585.

LAMIELL, J.M., SALAZAR, F.G. & HSIA, Y.E. (1989). Von Hippel-Lindau disease affecting 43 members of a single kindred. *Medicine*, **68**, 1.

LI, F.P., MARCHETTO, D.J. & BROWN, R.S. (1982). Familial renal cell carcinoma. *Cancer Genet. Cytogenet.*, **7**, 271.

LYONS, A.R., LOGAN, H. & JOHNSTON, G.W. (1977). Hypernephroma in two brothers. *Br. Med. J.*, **1**, 816.

MAHER, E.R., YATES, J.R.W. & FERGUSON-SMITH, M.A. (1990*a*). Statistical analysis of the two stage mutation model in von Hippel-Lindau disease and in sporadic cerebellar haemangioblastoma and renal cell carcinoma. *J. Med. Genet.*, **27**, 311.

MAHER, E.R., YATES, J.R.W., HARRIES, R. & 4 others (1990*b*). Clinical features and natural history of von Hippel-Lindau disease. *Quart. J. Med.*, (in press).

MAHER, E.R., BENTLEY, E., YATES, J.R.W & 8 others (1990*c*). Mapping of von Hippel-Lindau disease to chromosome 3p confirmed by genetic linkage analysis. *J. Neurol. Sci.*, (in press).

MAHER, E.R., BENTLEY, E., YATES, J.R.W., BARTON, D., AFFAR, N.A. & FERGUSON-SMITH, M.A. (1990*d*). Linkage analysis of von Hippel-Lindau disease (meeting abstract). *Am. J. Hum. Genet.*, A190.

MATHIESON, P.W. (1986). Renal carcinoma with a strong family history. *Br. J. Urol.*, **58**, 458.

MCLAUGHLIN, J.K., MANDEL, J.S., BLOT, W.J., SCHUMAN, L.M., MEHL, E.S. & FRAUMENI, J.F. (1984). A population-based case-control study of renal cell carcinomas. *J. Natl Cancer Inst.*, **72**, 275.

MELMON, K.L. & ROSEN, S.W. (1964). Lindau's disease. *Am. J. Med.*, **36**, 595.

NAYLOR, S.L., MARSHALL, A., HENSEL, C., MARTINEZ, P.F., HOLLEY, B. & SAKAGUCHI, A.Y. (1989). The DNF1552 locus at 3p21 is transcribed in normal lung and small cell lung cancer. *Genomics*, **4**, 335.

PATHAK, S., STRONG, L.C., FERRELL, R.E. & TRINDADE, A. (1982). Familial renal cell carcinoma with a 3:11 chromosome translocation limited to tumor cells. *Science*, **217**, 939.

PEARSON, H.H. (1969). Familial renal tumours. *Aust. NZ J. Surg.*, **38**, 333.

PILEPICH, M.V., BERKMAN, E.M. & GOODCHILD, N.T. (1978). HLA typing in familial renal cell carcinoma. *Tissue Antigens*, **11**, 487.

REEDY, E.R. (1981). Bilateral renal cell carcinoma-unusual occurrence in three members of one family. *Br. J. Radiol.*, **54**, 8.

RICHES, E. (1963). On carcinoma of the kidney. *Ann. R. Coll. Surg. Engl.*, **32**, 201.

RUSCHE, C. (1953). Silent adenocarcinoma of the kidney with solitary metastases occurring in brothers. *J. Urol*, **70**, 146.

SEIZINGER, B.R., ROULEAU G.A. OZELIUS, L.J. & 28 others (1988). Von Hippel-Lindau disease maps to the region of chromosome 3 associated with renal cell carcinoma. *Nature*, **332**, 268.

SHIMIZU, M., YOKOTA, J., MORI, N. & 4 others (1990). Introduction of normal chromosome-3P modulates the tumorigenicity of a human renal cell carcinoma cell line Ycr. *Oncogene*, **5**, 185.

SMITH, S.J., BOSNIAK, M.A., MEGIBOW, A.J., HULNICK, D.H., HORII, S.C. & RAGHAVENDRA, B.N. (1989). Renal cell carcinoma: earlier discovery and increase detection. *Radiology*, **170**, 699.

SOLOMON, D. & SCHWARTZ, A. (1988). Renal pathology in von Hippel-Lindau disease. *Hum. Pathol.*, **19**, 1072.

STEINBERG, S.M., BRODOVSKY, H.S. & GOEPP, C.E. (1972). Renal cell carcinoma in mother and daughter. *Cancer*, **29**, 222.

TEYSSIER, J.R., HENRY, I., DOZIER, C., FERRE, D., ADNET, J.J. & PLUOT, M. (1986). Recurrent deletion of the short arm of chromosome 3 in human renal cell carcinomas: shift of the c-*raf* locus. *J. Natl Cancer Inst.*, **77**, 1187.

TORY, K., BRAUCH, H., LINEHAM, M. & 9 others (1989). Specific genetic change in tumors associated with von Hippel-Lindau. *J. Natl Cancer Inst.*, **81**, 1097.

VALLETEAU DE MOUILLAC, M., GANASIA, R., HORS, J., LETEXIER, A. & MORIN, M. (1974). Cancer du rein familial et systeme H.L.A. *Nouv. Presse Med.*, **3**, 1539.

WANG, N. & PERKINS, K.L. (1984). Involvement of band 3p14 in t(3:8) hereditary renal carcinoma. *Cancer Genet. Cytogenet.*, **11**, 479.

YOSHIDA, M.A., OHKASHIKI, K., OCHI, H. & 5 others (1986). Cytogenetic studies of tumour tissue from patients with non-familial renal cell carcinoma. *Cancer Res.*, **46**, 2139.

ZBAR, B., BRAUCH, H., TALMADGE, C. & LINEHAM, M. (1987). Loss of alleles of loci on the short arm of chromosome 3 in renal cell carcinoma. *Nature*, **327**, 721.